# Vicarious Posttraumatic Growth in Peer-Support Specialists: An Interpretive Phenomenological Analysis

**DOI:** 10.3390/bs15121673

**Published:** 2025-12-03

**Authors:** Taryn C. Greene, Joshua R. Rhodes, Skyla Renner-Wilms, Richard G. Tedeschi, Bret A. Moore, Gary R. Elkins

**Affiliations:** 1 Boulder Crest Institute for Posttraumatic Growth, Bluemont, VA 20135, USA; rich.tedeschi@bouldercrest.org (R.G.T.);; 2 Department of Psychology, Abilene Christian University, Abilene, TX 79699, USA; 3 Department of Psychology and Neuroscience, Baylor University, Waco, TX 76706, USAgary_elkins@baylor.edu (G.R.E.)

**Keywords:** personal growth, posttraumatic growth, vicarious posttraumatic growth, well-being, positive psychology, vicarious growth, secondary trauma, burnout

## Abstract

Vicarious Posttraumatic Growth (VPTG) is a critical yet underexplored phenomenon among trauma-focused helping professionals. While secondary trauma (ST), compassion fatigue, and burnout are widely recognized negative aspects of working with trauma survivors, less is known about the potential benefits of this work and its contributions to well-being. This qualitative study explored peer-support specialists’ perceptions of growth arising from indirect exposure to trauma and examined how these experiences relate to well-being. Using interpretative phenomenological analysis, researchers conducted semi-structured interviews with 13 participants, independently coded transcripts, and developed themes through consensus. Findings suggest VPTG may follow a similar path to Posttraumatic Growth (PTG), with participants reporting challenges to core beliefs, emotional distress, and transformative cognitive-emotional shifts that facilitated growth across domains that appear to mirror the five PTG domains. Outcomes of working with trauma survivors extended beyond the PTG domains to include compassion satisfaction, hope, expanded coping skills, and improved mental health. Taken together, these findings illustrate the participants’ subjective experiences of both challenge and transformation through their work with trauma survivors, offering preliminary insight into how indirect trauma exposure may contribute to VPTG and well-being.

## 1. Introduction

### 1.1. Theoretical Background

Despite the well-documented challenges associated with working with trauma survivors, a growing body of research highlights that many trauma-focused helping professionals also experience Vicarious Posttraumatic Growth (VPTG)—positive psychological changes that arise from indirect exposure to trauma (Manning-Jones et al., 2015; Barrington & Shakespeare-Finch, 2012). Indeed, working with trauma survivors often involves a significant struggle characterized by heightened distress. Secondary trauma (ST) is an umbrella term describing these negative experiences, encompassing overlapping concepts like secondary traumatic stress (STS), vicarious traumatization (VT), compassion fatigue (CF), and burnout. Some researchers suggest this combination of negative reactions to indirect trauma exposure (ITE) can create conditions placing helping professionals at risk for deteriorating well-being and decreased effectiveness in working with trauma survivors (Leung et al., 2023). However, research also indicates that experiences of growth frequently coexist alongside distress, underscoring a more complex understanding of well-being as something that can emerge through, not apart from, adversity (Dekel et al., 2012; Shakespeare-Finch & Lurie-Beck, 2014).

While ITE has been shown to elicit emotional distress in individuals, there is evidence indicating this distress may also act as a catalyst for VPTG (Deaton et al., 2023; Manning-Jones et al., 2015; Barrington & Shakespeare-Finch, 2012). A systematic review reported more than 70 percent of trauma counselors described growth experiences in response to ITE (Deaton et al., 2023). Yet, studies of the relationship between ST and VPTG report inconsistent findings. Some studies found a significant positive relationship between ST and VPTG (Hamama-Raz & Minerbi, 2019; Shiri et al., 2008), others found a negative relationship (Măirean, 2016; Ogińska-Bulik, 2018), and still others detail the absence of a relationship between the two variables (Abu-Sharkia et al., 2020; Hamama-Raz et al., 2021). These inconsistencies highlight the need to integrate perspectives on both distress and growth, examining how they may unfold together within individuals exposed to indirect trauma.

Based on existing PTG-focused research, the benefits of PTG often offset distressing aspects of traumatic experiences, and this may also be true during the experience of VPTG. For example, research has identified PTG as an adaptive outcome that can co-occur with posttraumatic stress disorder (PTSD) symptoms, even moderating the effects of Posttraumatic Stress symptoms and depression (Morrill et al., 2008; Ruini et al., 2013). Additionally, positive associations have been observed between PTG and increased well-being (Pięta & Rzeszutek, 2022; Liu et al., 2020). It follows that experiences of VPTG may similarly buffer the deleterious effects of secondary trauma. Importantly, posttraumatic growth (PTG) does not occur in the absence of struggle; rather, it often develops alongside continued symptoms of distress (Shakespeare-Finch & Lurie-Beck, 2014). This interplay between pain and transformation represents a defining feature of the PTG process and provides a critical framework for examining how vicarious forms of growth may unfold in those who work closely with trauma survivors. Although prior research has explored statistical links between secondary trauma and vicarious growth, few studies have captured the lived experience of how these two processes coexist within individuals.

Furthermore, although prior studies suggest that some aspects of VPTG may resemble the processes described in PTG theory, little is known about whether the pathways of change in VPTG align conceptually with the five domains of PTG. To date, most studies of Vicarious Posttraumatic Growth (VPTG) have sought to quantify it as an outcome, yet few have examined VPTG as a process (Manning-Jones et al., 2015; Deaton et al., 2023; Tsirimokou et al., 2023). This represents a clear gap in understanding how growth and well-being develop in the context of ongoing struggle with ITE. This gap highlights the need for qualitative inquiry that can illuminate how those working closely with trauma survivors interpret and make sense of their own internal struggle and the potential growth arising from this work. In light of these conceptual and empirical gaps, the current investigation was designed to address these issues by examining how ST and VPTG are experienced and interpreted within the context of ongoing exposure to others’ trauma.

### 1.2. The Present Study

The present study explores: (a) how peer-support specialists experience VPTG and whether their accounts suggest processes or outcomes that conceptually parallel the five domains of PTG; (b) how these experiences intersect with secondary trauma (ST); and (c) how they relate to perceived well-being and adjustment following indirect trauma exposure. Given the limited research on VPTG, particularly in relation to ST, this study sought to examine the lived experiences of VPTG and ST within the occupational and relational context of peer-support.

The target sample comprised peer-support specialists who were also public safety personnel (PSP), including military personnel, military veterans, and first responders, who deliver PTG-focused programming to other PSP who have experienced trauma. PSP represent a population with disproportionately high exposure to traumatic events and elevated risk for suicide (Martinmäki et al., 2021). Although research on PSP has largely emphasized PTSD, burnout, and related pathology, considerably less attention has been given to their experiences of PTG and VPTG. By focusing on both risk and growth in this population, the study sheds light on how vicarious posttraumatic growth experiences can contribute to well-being in groups often defined primarily by vulnerability to stress and trauma. This sample provides a unique opportunity to learn from individuals who are not only at heightened risk for psychological trauma, but who also occupy a distinctive role in fostering growth in others and in experiencing growth themselves.

In summary, while theoretical parallels between PTG and VPTG have been proposed, the field lacks detailed, phenomenological accounts of how these processes unfold in lived experience. The present study aims to address this gap by examining how peer-support specialists, who routinely engage with indirect trauma, describe and make meaning of both struggle and growth. By examining these experiences via an interpretative phenomenological framework, this study contributes to theory by illuminating how processes of meaning-making, growth, and well-being may develop in the presence of ongoing distress. Centering participants’ own interpretations allowed for a rich understanding of how VPTG, secondary trauma, and related outcomes emerge among peer-support specialists frequently exposed to indirect trauma. The study’s insights aim to inform both theory and practice, enhancing conceptual understanding of vicarious growth processes and supporting researchers, program developers, and practitioners who seek to promote well-being among those who serve trauma-exposed populations.

## 2. Materials and Methods

### 2.1. Participants

A qualitative study was conducted among a purposive sample to collect and analyze data from thirteen (13) peer-support specialists who work at a Posttraumatic Growth-based program for trauma survivors at a nonprofit organization. The program is run by peer-support specialists who have previously completed the program themselves, and most of whom are prior military or first responders. Individuals served by the program are military members, veterans, and first responders who report struggling in the aftermath of traumatic events. Eligibility criteria specified that individuals must be at least 18 years of age, be employed as a peer-support specialist in the program, and endorse having an experience of VPTG during their time in this role.

The aim of this study was not to estimate the prevalence or frequency of self-reported VPTG among peer-support specialists, but rather to develop a richer understanding of its structure and meaning when it is experienced among this sample. Because Interpretive Phenomenological Analysis (IPA) design emphasizes an in-depth exploration of participants’ lived experiences within a relatively homogenous group, participants were purposefully recruited based on their self-identification of having experienced some degree of VPTG. The inclusion criteria were designed to ensure participants could meaningfully reflect on the experience of growth in this context, thereby supporting the study’s analytic depth and interpretive coherence.

### 2.2. Participant Recruitment and Selection

A recruitment message was shared with staff who might fit study criteria. Peer-support specialists who contacted study investigators were screened to confirm they met inclusion criteria and were available for a 60–90-minute recorded interview during the targeted data collection period of September 2024. Prospective participants who met all eligibility requirements were walked through a consent process and emailed an electronic consent form to sign and return. Once consent was obtained from the prospective participants, their interviews were scheduled on a rolling basis. Participants were given the option to withdraw at any point leading up to and during the interview process.

### 2.3. Data Collection

Each participant completed one semi-structured, one-on-one interview with an investigator using an interview protocol designed in accordance with principles of interpretative phenomenological analysis (IPA; Smith et al., 2009; Willig, 2013). This interview protocol focused on open and exploratory questions aimed at uncovering the individuals’ lived experiences. Questions were crafted to specifically examine the experiences of ST and VPTG with a particular focus on how and in which way the experiences were connected to mental health and well-being.

Individual interviews were conducted in English, using Zoom Workplace (Version 6.2.11), and were audio recorded. Recordings from the interviews were then transcribed using NVivo (Version 15). Following the initial transcription, a researcher compared the transcript with the original audio file to correct any errant transcriptions. In the event of a potentially errant transcription that was not coupled with clear audio, a second researcher was consulted to arrive at an agreed upon transcription.

### 2.4. Data Analysis

An IPA approach was used to analyze the collected data. This approach is well-suited for exploring how individuals in the sample make sense of their lived experiences while allowing existing research and theoretical frameworks to inform interpretation. In accordance with a structured IPA methodology, all transcripts were analyzed with an idiographic approach and were reviewed on multiple occasions before moving to cross-case analysis. Each transcript was independently analyzed by two researchers, one an experimental psychologist and the other a health psychologist, both with backgrounds in trauma and posttraumatic growth. During this independent analysis phase, each researcher created annotations and codes for concepts that emerged from the transcripts. Once all transcripts were completed, researchers met to compare interpretations, identify areas of convergence and divergence, and resolve discrepancies by consensus, to ensure that final codes accurately reflected participants’ intended meanings.

Throughout the analytic process, both researchers engaged in reflexive bracketing (Tufford & Newman, 2010) to acknowledge and examine their assumptions about trauma and vicarious posttraumatic growth. Reflexive dialogue between the two researchers shaped the analytic process and frequently led the team to revisit earlier interpretations of the transcripts. These discussions focused on examining how assumptions informed by the researchers’ prior knowledge of PTG might inadvertently overemphasize growth-oriented meanings in these data. Such reflexive exchanges resulted in concrete analytic shifts, including deliberate efforts to distinguish between typical growth and growth specifically related to indirect trauma exposure, as well as a sustained emphasis on capturing the full complexity of participants’ accounts, attending equally to experiences of distress, neutrality, and growth. Analytic notes and documentation within the coding and collaboration software were maintained to provide an audit trail of evolving thoughts, dialogue, and thematic development. Team discussions served as peer debriefings to ensure interpretations remained grounded in participants’ accounts. Deviant or disconfirming cases were deliberately examined to assess whether they represented contradictions or alternate expressions of similar outcomes.

The coding framework was informed by an existing conceptualization of Posttraumatic Growth. When emergent themes aligned with established PTG dimensions, these intersections were noted; however, themes were not forced into preexisting categories and novel themes were able to be developed inductively. This process led to the identification of overlapping and distinct patterns of VPTG reported by participants. Initial codes were iteratively refined and condensed through cross-case comparison to create final, higher-order themes supported by multiple participants. As coding progressed, several early descriptive codes were refined as recurring patterns became clearer across transcripts. For example, an initial broad code labeled deliberate rumination was later differentiated into two more specific subcodes, (1) deeper self-reflection and (2) schema change, to capture distinct cognitive processes described by participants.

Two transcripts diverged from the majority in different ways. One participant emphasized an experience of resilience along with distress and growth in response to ITE, and still described growth in several VPTG domains. The researchers noted this divergence while ensuring that the participant’s VPTG-related material remained represented. Another participant reported more distress than growth but likewise described several VPTG domains; in this case, the distress was incorporated into themes focused on distress, as it reflected patterns seen across the wider sample.

These cases prompted the researchers to consider whether additional codes were warranted to better conceptualize ST and VPTG within the sample. Ultimately, the research team revisited all transcripts and codes to confirm that the final themes adequately accommodated such variation without oversimplifying the analytic narrative. Although the study did not seek theoretical saturation, analysis of all thirteen interviews demonstrated strong convergence across the majority of cases. Representative quotations were selected to illustrate each theme, ensuring findings were deeply grounded in participant’s voices and reflectively interpreted within the context of VPTG.

## 3. Results

Thirteen (13) peer-support specialists completed semi-structured interviews with study personnel. Participants were predominantly male (*n* = 10) and ranged in age from 32 to 59 (M = 49.0, SD = 9.44). On average, participants had been working as peer-support specialists for 2.54 years (SD = 1.81) and worked as peer-support specialists for an average of 21.31 PTG-based program sessions (SD = 13.56) in total. Eleven (11) participants were U.S. military veterans from the branches of Marine Corps, Army, Navy, and Air Force (*n* = 3, *n* = 6, *n* = 1, *n* = 1, respectively) who served for an average of 17.95 years (SD = 7.98). The remaining participants (*n* = 2) were an emergency medical technician (EMT)/first responder with 10 years of service and a civilian program-curriculum subject matter expert.

In keeping with IPA’s emphasis on idiographic nuance, the analysis sought to capture both convergent and divergent elements of participants’ experiences. While many described growth-oriented changes, most also reported substantial distress, doubt, or periods of stagnation, particularly early in their peer-support work. Attention was paid in the analysis to capturing both elements and to reflect the full breadth and complexity of participants’ experiences with the impacts of ITE.

**Figure 1:**
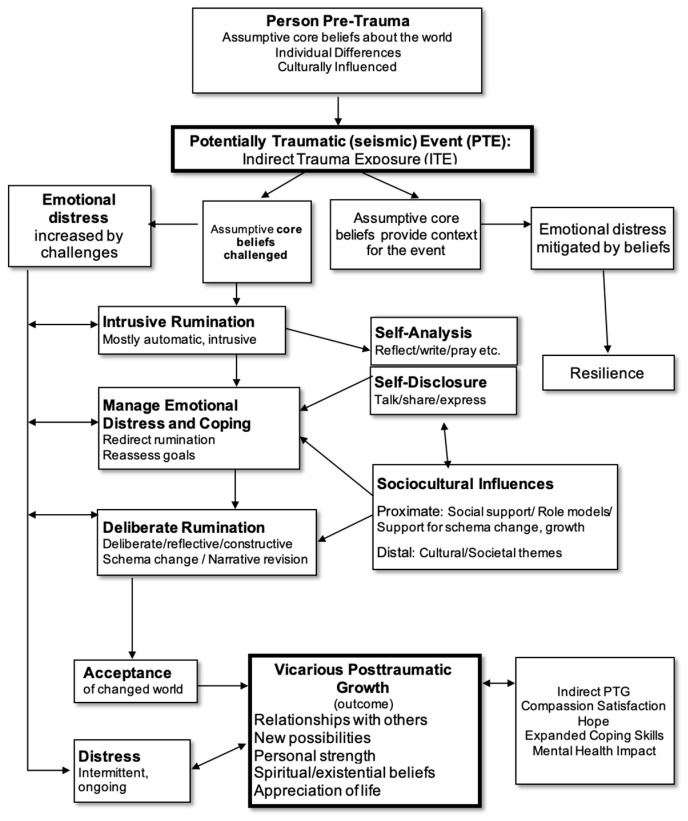
Proposed Model of the VPTG Process: Adapted from Tedeschi et al. (2018).

Through IPA, seven (7) superordinate themes and 23 subthemes related to the experiences of ST and VPTG were identified. These themes were grouped into two overarching categories and are presented accordingly: VPTG Process themes and VPTG Outcomes themes. Each theme, its description, subthemes, and representative quotations from participants are presented below. Following each quotation, a de-identified participant ID number ranging from 1 to 13 is provided in parentheses to reference which participant is responsible for each representative quotation provided (i.e., the participant ID number corresponds to the quotation preceding it). A proposed model (Figure 1) of the VPTG pathway is presented below, which represents an adaptation of the existing model of Posttraumatic Growth proposed by Tedeschi et al. (2018). This model is offered as a preliminary conceptualization of how the themes expressed by participants in this sample may map onto an established framework of a similar construct (PTG). While this proposed model provides a useful foundation for understanding VPTG as experienced by peer-support specialists, additional research and data would be required to evaluate its generalizability and empirical validity.

In the figure, elements that diverge from the original PTG model are bolded. These modifications include specifying indirect trauma exposure (ITE) as the initiating “potentially traumatic event,” identifying the primary outcome as Vicarious PTG rather than PTG, and incorporating an additional box adjacent to the VPTG Outcomes box to represent the associated outcomes reported by participants in this study, which differ from those outlined in the original PTG model.

### 3.1. VPTG Process Themes

The following themes represent areas of the VPTG process described by the peer-support specialists.

*Superordinate Theme 1. Trigger Events for Vicarious Posttraumatic Growth (VPTG)*

Participants described two conditions that, in their accounts, appeared to serve as catalysts for VPTG: (1) experiencing ITE as potentially traumatic and (2) encountering challenges to their assumptive core beliefs. These perceived catalysts, along with the subsequent aspects of the VPTG process, are outlined below.

**Indirect Trauma Exposure as a Potentially Traumatic Event.** Peer-support specialists reported indirect trauma exposure being potentially traumatic in two ways.

First, hearing details of traumatic stories shared by participants brought their own past personal traumas to the surface. One participant said,

*Hearing these stories called on the stories that I hadn’t thought about or processed myself and their grief or pain connected with my very real grief and pain.*

(6)

Second, participants’ disclosures from trauma survivors about abuse and/or sexual abuse, especially in women and children, were particularly hard to hear. Two participants disclosed,

*To see women that were violated in that way, it still hurts me.* (6) *Anything that had to do with having the innocence taken away from a child or like verbal and emotional abuse, abandonment, stuff like that. Because those were things that I had never really addressed. Maybe in like small doses… when I’d hear those things, it would just kind of take me into that place.* (4)

**Assumptive Core Belief Challenged.** Participants reported challenges to core beliefs via indirect trauma exposure akin to the experience of trauma happening directly to a person and challenging their core beliefs about the self, others, and the world. One participant said,

It became, I would say, morally challenging…So I shifted from this, “Holy sh*t. I can’t believe I’m hearing this. I feel so bad for this person.” And then it became, “Wow, what’s really going on in the world?”

(5)

*Superordinate Theme 2. Distressing Internal Responses*

Peer-support specialists reported various forms of distressing internal responses following trigger events. Many noted these responses were particularly intense and frequent during initial months and years of working with trauma survivors—a phase that will be referred to as the “acclimation period.” This potential acclimation period, along with a subsequent “thriving period” observed in the sample, are discussed in greater detail later in the paper. Importantly, several participants described these early experiences as overwhelming and, at times, destabilizing, reflecting substantial distress alongside any later growth.

**Emotional Distress.** Participants in the sample reported emotional distress arising in response to trigger events including increased heart rate, feelings of anxiety, flashbacks, sadness and depression, trouble sleeping, and nightmares.

The nature and intensity of the emotional distress can be understood within the framework of PTSD/ST. For example,

*“So there has been a few times during the struggle portion where I kind of found myself zoning off in their story and started reflecting on my own. One of them even brought back the smells, because that’s all he was talking about. And it took me a couple of days to stop thinking about it to where I wasn’t actually smelling those smells again”.* (10) *“I had a set of severe nightmares that happened for a two week period…it was like this two week period where I was like, ‘Oh my God. I don’t know if I can do this job if this is what was going to happen’”* (1)

While many participants later described increases in coping capacity and reductions in distress, these accounts illustrate that the experience of working with trauma survivors was not uniformly positive nor immediately growth-oriented for everyone. Distress and doubt were prominent for some participants, particularly early in their work, and for a few, these feelings recurred intermittently even during periods they associated with growth.

**Intrusive Rumination.** Some participants reported experiencing intrusive thoughts in response to ITE. Two participants said,

*“That’s probably the main thing…unwanted thoughts.* (6) *It would trigger memories of events that I either hadn’t thought of for years and years…I was having intrusive thoughts. It was taking me back to times and places that I didn’t want to revisit”.* (1)

*Superordinate Theme 3. VPTG-Oriented Internal Responses*

For many participants, trigger events and distressing internal responses were described as giving rise to VPTG-oriented internal responses.

**Self-Analysis.** Participants reported two aspects of self-analysis, described here.

***(a)***  ***Growing Self-Awareness.*** A growing self-awareness of internal distress was triggered by hearing stories of trauma from program participants. One participant disclosed,
  *“There are moments that sort of tug at me, but… my practices of regulation have gotten so much stronger so I can identify, ‘Hey, I know what this is. This is some anxiety rolling in.’”*
  (7)
***(b)***  ***Introspection-Focused Action.*** Participants noted an evolving ability to use this self-awareness of distress as a cue to engage in introspective activities. For example,
  *“I did not expect this kind of emotional reaction. So, what I’ve essentially done is investigated that. It’s not something that I set aside anymore or push down or deny. It’s something that I investigate. I sit with it, and I have what I call a curiosity journal. I’ll pull that out and start writing down what emotions I’m dealing with and why am I having those emotions”*
  (1)

**Self-Disclosure**. Participants reported engaged in constructive self-disclosure when experiencing internal distress, such as sharing their feelings and challenges with peers, friends, and family. Self-disclosure is intentionally integrated into both the curriculum and the overall experience of the PTG-based program. One participant disclosed,

*“Before [being a peer-support specialist] I would just kind of put the mask on, continue about my day and continue just pile on myself in my rucksack and just forgetting about it. Now, I know it’s not okay to do that. So…I’m more open to having that talk with someone if I need to have that talk with someone”.*

(10)

**Managing Emotional Distress.** Participants emphasized the importance of regulation-focused activities as a way to manage internal distress and to set conditions for more intentional reflection. One participant disclosed,

*“A lot of times at the end of the day when I go back to my room, I’ll do a meditation or I will do a breathing exercise or something if I’m feeling really heavy from the day so that I’m not carrying that into the next day.*

(3)

**Deliberate Rumination.** Almost all participants described engaging in deliberate rumination in response to internal distress. Several described viewing distress as a signal to “go into their cave”, a metaphor for introspection inspired by Joseph Campbell’s quote, “The cave you fear to enter holds the treasure you seek,” which is included in the program’s curriculum. This metaphor appears to reflect a shared belief among the group that internal distress serves as an invitation for deeper self-reflection and growth.

***(a)***  ***Deeper Self-Reflection***
  *“There was one particular class, a female class, where a lot of things really hit home for me. Even though I was kind of processing things throughout the week, I had made some notes for myself. Like, ‘When you get home, you need to deep dive into these things.’ So I took some time during those two days to really deep dive and process those emotions… I used that time to really dissect those emotions too.* (3) *We teach ‘the cave to fear to enter holds the treasure you seek.’ Every month I’m going into my cave and I’m looking at something different and sharing that with students…in order to help someone who’s struggling, I have to get into my cave. I can’t give them wisdom if I don’t go in there”.* (2)
***(b)***  ***Schema Change***
  Participants described how entering their metaphorical cave allowed them to deliberately reflect on their internal world and change their own deeply held schemas. Examples of participants’ changed worldviews are,
  *“It’s [working with trauma survivors] opened my eyes to hundreds of new perspectives on hundreds of different things that I’ve experienced in my life, which has helped me kind of make sense of those things.* (1) *One of the core beliefs that I’ve always maintained from the time I was a child is that we can change the world. And…in order for…it to happen on an individual basis. The change has to happen on an individual basis. And so, do I believe we can change the world? I do, but no longer as simple as I believed it could be. So that’s a core belief that has changed”.* (6)

**Acceptance.** Participants described acceptance of new perspectives that came about through the deliberate *rumination process. One participant disclosed,*

*“I see how bad life can be. I see how evil humans can be. How hurtful humans can be. And then watching them change and grow gives me…every [program]…gives me a newfound appreciation for life for the large and small things in life”.*

(8)

*Superordinate Theme 4. Positive Sociocultural Influences*

**Safe Space.** Participants reported the importance of trust, safety to process emotions, and mutual support from other staff, in being able to engage in VPTG-oriented internal responses. Two participants said,

*“Being in a safe and trusting environment where I’ve got [other staff members] that I trust implicitly—and they do the same with me and we create an environment for the students as well—allows me to, I don’t know, dig into some of those emotions that I would have previously ran away from.* (1) *I feel like building that safe and trusting environment is just as important for the [staff members] as it is for the students”.* (3)

*Superordinate Theme 5. Acclimation and Thriving Period*

**Acclimation Period.** Many peer-support specialists recalled an initial period of adjustment when working with trauma survivors, during which they acclimated to repeated ITE. During this phase, they reported finding the work particularly challenging and described the emotional investment as deeply exhausting.

*“I think in the beginning, my biggest symptom was that I would be emotionally exhausted after program because there was a lot of things that I would think about… My first couple of classes, I definitely needed more time to recover from those things because I just felt just physically, mentally, emotionally drained from those things”.*

(9)

**Thriving Period.** Most peer-support specialists described reaching a place of thriving in their work with trauma survivors, where they gradually became less overwhelmed by distressing internal responses to ITE. They described distressing responses becoming less frequent and less intense and that they felt more equipped and knowledgeable about what to do when distress arose. Many also described that the thriving period became possible because of the work they did during the acclimation period to come to terms with past personal traumas and the acceptance they felt for the changes in their worldviews. Many reported that internal distress persisted even during the thriving period, remaining an ongoing part of their work with trauma survivors, but they felt better able to manage it. For example,

*“So in the beginning it was difficult, it was harder than it is now for me”.* (5) *Now, any time something arises for me, that doesn’t go away, I’m talking about it, I’m writing it down, I’m crying about it. I’m addressing it, I’m doing what I can to process it so that it doesn’t cause me to go sit down again. And so as I hear trauma survivors today, it doesn’t call back out my own trauma. Those experiences are there. I remember them, but it’s not as palpable. I don’t feel it within myself as much as I did”.* (6)

It is important to note that this perceived movement from acclimation to thriving reflects participants’ interpretive accounts within this specific context and does not constitute empirical evidence of a linear or universal developmental trajectory. Some participants’ accounts reflected more cyclical trajectories, and a few did not describe the acclimation-to-thriving pattern at all, highlighting the idiographic nature of IPA even though this trajectory was common among the majority.

### 3.2. VPTG Outcomes Themes

The following theme represents the outcome, VPTG, as described by the peer-support specialists.

*Superordinate Theme 6. Vicarious Posttraumatic Growth.*

By nature of the study’s inclusion criteria, all peer-support specialists reported an experience of VPTG. This general experience of VPTG was best captured by the statements,

*“I have a choice when I have a struggle, when you tell me something that strikes something within me, I now have an opportunity to grow …. That doesn’t mean it’s not going to hurt because much of it is going to hurt. But if I approach it from a place that says what’s available for me to take away from this…what’s available to glean from it.* (7) *I can’t put a finger on it. I think it’s not always one trauma but a death by a thousand cuts. I think growth by a thousand little seeds planted through a thousand different people for sure”.* (2)

Further, participants reported VPTG using the verbiage of the five specific domains in a model of PTG presented by Tedeschi and Calhoun (Tedeschi et al., 2018). In order of prevalence, participants reported VPTG in the areas of relating to others, appreciation of life, spiritual-existential change, personal strength, and new possibilities.

**Relating to Others.** All thirteen peer-support specialists reported changes in relating to others as a result of secondary trauma exposure, especially deepening of relationships. This change took the form of being more comfortable being vulnerable in relationships and changing the framework of their own needs resulting in increased interpersonal transparency. These reports are best illustrated by,

*“… understanding why I’m feeling the emotions that I’m feeling, and then starting to process through those have led to conversations with my family and deepening a relationship with my mother, for example, who I didn’t speak to for years because I’m able to understand a little bit better what she was dealing with at the time.* (1) *It has helped me be able to be more transparent and more honest with other people, especially when it comes to my needs”.* (3)

**Appreciation of Life.** Participants routinely discussed increasing appreciation of life. ITE seemed to highlight the potential for loss, as well as the remaining and resulting gifts, that happen in a multitude of areas or people. For example,

*“We do hear a whole mix of different things that people have experienced. And I just think that… well again, it goes back to the appreciate appreciation for life for me. Just appreciating what I’ve had in my lifetime that maybe someone else didn’t”.*

(11)

**Spiritual-Existential Change.** Reports on spiritual-existential change were largely focused on personal shifts in perspective. This shift, at times, indicated a growing interest in spiritual dimensions of the human experience and, at other times, reflected an evolving understanding of the self and its relation to existential issues of purpose and identity. Two participants said,

*“There’s something that is happening with me in the way of spiritual existential change. I’m not a religious person by nature … That’s just not where I’m at. But as I watch participants come through, I find myself sort of getting hung on where they’re connecting to this universe and what they’re seeing is bigger than themselves. It’s an interesting concept to me.* (7) *Just watching that process happen each month is something that is no longer a question of “maybe” or “A few of us on Earth has this potential,”—it’s we all have the potential to separate our experiences from our actual spirit and ourselves. And that spiritual and existential change is how I understand it. Like you’re beginning to hold on to more of what you are or your being and less of what you’re being has gone through”.* (6)

**Personal Strength.** Participants described increases in personal strength tied to witnessing an enduring human capacity to overcome adversity. Additionally, they seemed to use students’ stories of trauma and PTG as tools to reframe their own experiences, resulting in an increased sense of strength for having not only survived their own experiences, but having grown in the process. Individuals reported,

*“As I see them [the participants] grow and get stronger, I get stronger myself. Because I see that you can live through something so traumatic, so horrific. …just because I have the symptoms doesn’t mean that I don’t have inner strength. In fact, my ability to manage to live with those symptoms is an example of my inner strength”.*

(5)

**New Possibilities.** Participants described an increasing sense of being able to change things that seem to need changing, and of being able to embrace going in new directions because of their exposure to students overcoming trauma.

*“I get to see the contrast of what life could be or was for me at some point in time and really take stock of where I’m at today. Each program I come up with new goals for my future. I come up with ways that life could be better if I make simple decisions or changes each day.* (6) *And a lot of that is this from the conversations that I’m having with the students, where “Hey, I’m really struggling with this. What’s your take on this subject?” Those conversations lead to questions about myself and about my life and different avenues or ways that I can continue to grow on my journey”.* (1)

*Superordinate Theme 7. Associated Outcomes.*

The following theme represents other outcome associated with VPTG reported by the peer-support specialists.

**Indirect Posttraumatic Growth.** There were frequent reports of experiencing growth as a result of witnessing the PTG process unfolding for students, which served as inspiration and motivation. While it is difficult to determine whether this translates to actual growth within the peer-support specialists, reports include,

*“In a way it’s exciting because I know that on the other side of that trauma is something beautiful, something amazing. And getting to watch that grow and flourish and develop over even seven days”.*

(8)

**Compassion Satisfaction.** Reports of growth as a byproduct of watching others grow can be conflated with the phenomenon of compassion satisfaction. While participants did not use the verbiage of “compassion satisfaction” there were many instances in which the experience they reported has been interpreted as compassion satisfaction. An example is,

*“Being able to work with individuals to see their own self-worth to be able to see that they’ve got control and power to make significant change in their lives. I really wish I could find another word except rewarding, but that’s really what it is”.*

(1)

**Hope.** Participants frequently reported experiences of hope as a result of working with program participants. Two participants said,

*“now I see hope. Like when I see someone struggling, I know that when they learn how to appropriately struggle or struggle well, that there are going to be…I’m hopeful for them.* (5) *And so when I look at everything, I look at the totality of it. And to me, there’s such hope and the possibility of a brighter future”.* (8)

**Expanded Coping Skills.** Participants frequently reported expanded coping skills as a result of working with students, which often involved increases in emotional awareness, emotional regulation, and self-efficacy towards being able to cope. For example,

*“I still have certain thoughts and feelings. What’s different now is I don’t react to them… So is it okay to get angry? Yes. Is it okay to be anxious about certain things? Yeah, absolutely. Everybody gets those things…but my response is much different than how I used to react before. It’s tremendously different.”*

(9)

**Mental Health Impact.** Reports on mental health impact were net-positive, with participants reporting a seeming acclimation period in which the secondary trauma exposure resulted in heightened emotional distress that was gradually eliminated, resulting in a sense of freedom and clarity. Two participants said,

*“I really do feel free. Not only from what I used to carry, but from the fear that something else will happen and that I’ll have to carry that all over again. I know the process of healing or getting myself out of that.* (6) *It’s made me healthier than I would have been otherwise… The clarity that I have on how our minds work and the things we tell ourselves and how those affect our thoughts and actions.”* (5)

## 4. Discussion

This qualitative study used an interpretative phenomenological analysis (IPA) approach to examine the experiences of thirteen peer-support specialists, the majority of whom were military personnel and first responders, and who also had experiences facilitating a PTG-based training program for other military personnel and first responders. Individual interviews were conducted and analyzed to understand how these peer-support specialists experienced secondary trauma (ST) and vicarious posttraumatic growth (VPTG) because of indirect trauma exposure (ITE). As with all interpretative work, these findings are shaped by both researcher and participant positions. The research team, comprising psychologists with backgrounds in trauma and posttraumatic growth, approached the analysis through reflexive bracketing and peer debriefing to examine assumptions and remain grounded in participants’ accounts. It is possible that the involvement of the participants in a PTG-focused peer-support program that aims to facilitate growth and strength in the aftermath of trauma may have influenced how the participants made meaning of growth. The PTG-centered culture of the setting may have amplified the participants’ tendency to interpret struggle through the framework of growth, even when their experiences included ambivalence or ongoing distress. The present findings should be understood as context-specific interpretations of how these PTG-trained peer-support specialists make meaning of their experiences of secondary trauma and potential growth.

The study identified seven superordinate themes and 23 subthemes related to these experiences in the sample, representing the researchers’ interpretation of participants’ accounts of ST and VPTG. These themes were further divided into two main categories, The VPTG Process (encompassing 5 superordinate themes and their associated subthemes), and VPTG Outcomes (encompassing 2 superordinate themes and their associated subthemes). The findings, summarized in Figure 1 and summarized below illustrate the VPTG process as described by the sample group. Together, these themes highlight how peer-support specialists described experiencing profound personal growth, transformation, increased well-being, and fulfillment through their work with trauma survivors as well as struggle, including symptoms of secondary trauma.

### 4.1. The VPTG Process

Peer-support specialists described experiencing distress and growth in response to Indirect Trauma Exposure (ITE) through a process that may mirror the PTG process (See Figure 1). Key themes summarizing these experiences included:

1. **Trigger Events for VPTG**—Indirect Trauma Exposure (ITE) presented challenges to core beliefs about the self, others, and the world, initiating the process of VPTG.
2. **Distressing Internal Responses**—Exposure to ITE and associated challenges to core beliefs caused emotional distress (e.g., anxiety, sadness, nightmares) and intrusive thoughts, which together can take the form of symptoms of ST.
3. **VPTG-Oriented Internal Responses**—As individuals navigated distress, VPTG-Oriented Internal Responses emerged, characterized by self-analysis, self-disclosure, emotional regulation practices, deliberate rumination, and ultimately, acceptance of the way things had changed, including acceptance of positive changes in the aftermath of trauma.
4. **Positive Sociocultural Influences**—These cognitive and emotional shifts were promoted by the presence of trust and emotional safety in relationships which provided a foundation for processing internal distress and integrating new insights.
5. **Acclimation and Thriving Period**—Participants described that, over time, they became more able to tolerate the emotional demands of working with trauma survivors and to engage more intentionally in their own growth and thriving as a result of indirect trauma exposure. The researchers characterized this progression as a shift from an “Acclimation Period”, marked by frequent and intense distress, to a “Thriving” period, characterized by increased capacity for constructive, growth-oriented practices and less overwhelming distress.

This progression appears to parallel elements of the PTG process described in individuals who have directly experienced trauma. The first four of these five themes can be understood as aligning conceptually with aspects of the foundational model of PTG presented by Tedeschi et al. (2018). Additionally, these findings build on existing PTG theory by illustrating how growth-related processes may occur not only following direct trauma but also through sustained, empathic exposure to others’ trauma. This extension of PTG theory highlights the relational and ongoing nature of PTG, adding nuance to a model traditionally centered on primary traumatic events directly experienced by the person. A distinction of the VPTG process appears to be in the fifth theme, whereby repeated exposure to the potentially traumatic events in the form of ITE allowed for the transition from an acclimation to a thriving period while exposure to potentially traumatic events continued. The acclimation period appears to encompass characteristics of ST, and then to evolve into the thriving period as the peer-support specialists make use of the very processes of PTG they are facilitating with students. This interpretation reflects the researchers’ understanding of patterns described by participants in this specific context and should be viewed as a tentative, narrative representation rather than a generalizable model of progression.

A further defining feature of the VPTG Process for the sample was the apparent expertise with which peer-support specialists engaged in VPTG-oriented internal responses when facing distress, a practice referred to by participants as “going into the cave.” Almost all participants discussed recognizing emotional distress as a signal to engage in deeper self-reflection rather than avoiding or suppressing their feelings. The phrase, drawn from Joseph Campbell’s well-known quote, “The cave you fear to enter holds the treasure you seek”, is an explicit part of the PTG program curriculum and appeared to serve as a guiding principle among the group of peer-support specialists interviewed. Within this framework, distress is not viewed as a disruptive force but as an invitation to explore and reconstruct core beliefs about the self and the world. For the participants these practices often involved journaling, conversations with trusted peers, or mindful contemplation, all of which facilitated cognitive restructuring. The specialists reported fundamental shifts in their perspectives because of engaging in these VPTG-oriented internal responses, including a greater sense of self-efficacy, increased emotional intelligence, and a reframing of trauma exposure as a transformative experience. Given their training and experience in facilitating PTG for others, participants demonstrated both conceptual familiarity with and intentional engagement in the PTG process. Their accounts reflected a willingness to apply the principles they teach, suggesting that their meaning-making was shaped not only by their personal experiences of distress but also by their deep immersion in a PTG-oriented cultural and institutional context.

### 4.2. VPTG Outcomes

Consistent with elements of PTG theory (Tedeschi et al., 2018), participants in this study spoke to both the process and the outcomes of VPTG. Two themes emerged in this realm: (1) The first theme, VPTG as an Outcome, illustrated how struggle and cognitive processing from ITE catalyzed growth in five areas congruent with the five domains of PTG: deeper relationships, greater appreciation for life, spiritual and existential development, increased personal strength, and, to a lesser extent, new life possibilities. (2) The second theme, Associated Outcomes, was characterized by reported benefits associated with the VPTG experience, such as drawing inspiration from witnessing PTG in others, feeling a sense of deep pride and joy in the work (e.g., compassion satisfaction), increased hope in the innate human ability to overcome hardship, expanded coping skills, and overall improvements in mental health over time.

Together, these themes highlight how peer-support specialists described experiencing profound personal growth, transformation, increased well-being, and fulfillment through their work with trauma survivors. While these professionals reported experiencing VPTG and associated benefits, as reported above, their work is not without significant emotional investment and struggle. The process that leads to VPTG involves facing the distressing realities of trauma, and this underscores the complexity of the work of assisting trauma survivors. Collectively, these findings suggest that VPTG may represent a relational extension of PTG, one that occurs through empathic connection, witnessing, and meaning-making rather than direct trauma experience. Taken together, these results contribute to theoretical understandings of PTG by positioning VPTG as a related, relational process that may unfold in similar ways to PTG, and that may be especially common within systems explicitly designed to promote growth.

## 5. Limitations and Future Directions

Further research is needed to deepen researchers’ collective understanding of how VPTG unfolds over time and to explore strategies that may enhance the likelihood of VPTG in those engaged in trauma-related professions. To build on the current findings, researchers should aim for replication of qualitative studies investigating the lived experiences of ST and VPTG with other samples as well as investigate the most appropriate measurement tools for assessing VPTG. Moreover, future studies should prioritize quantitative research examining relationships between ST and VPTG to clarify mechanisms underlying these phenomena, to understand the summative effects of each in the context of working with trauma survivors, and to identify factors that may facilitate or hinder VPTG and associated benefits in those exposed to the suffering of others.

A limitation of the current study lies in the difficulty of parsing out VPTG from typical growth. The nature of the study participants, peer-support specialists who have had previous training in PTG, may indicate that participants are primed for viewing even typical growth through the lens of PTG and therefore, VPTG. This intentional selection of participants familiar with PTG also means that accounts of growth may be more readily articulated here than among samples unfamiliar with PTG and should not be interpreted as representative of all helping professionals. Furthermore, the focus on a specific population, peer-support specialists who are predominantly military veterans and first responders may limit the generalizability to other trauma-focused professionals or to those outside structured PTG-based programs.

The positionality of the researchers as PTG-informed psychologists is itself a limitation, as it may have influenced interpretative decisions and the framing of themes despite the use of reflexive bracketing. This background may have shaped how elements of growth were recognized, emphasized, or organized within participants’ accounts.

Additionally, while analytic rigor was supported through peer debriefing and reflexive bracketing, the study did not include formal member checking or methodological triangulation, which may limit the interpretative depth and confirmability of findings. The interpretations presented here should be viewed as one contextually informed interpretation of participants’ accounts, grounded in an idiographic approach tied to this specific setting.

Additional limitations include a small sample size of thirteen participants, resulting in findings that may not fully capture the diversity of experiences among peer-support specialists. While Interpretative Phenomenological Analysis (IPA) emphasizes depth over breadth, a larger or more varied sample could provide additional insights. Finally, the retrospective nature of participant reports introduces the potential for recall bias in which their perceptions of distress and growth may have evolved over time, influencing their descriptions of such experiences. Overall, these limitations indicate that the findings should be understood as exploratory and illustrative rather than generalizable.

## 6. Conclusions

The findings of this study contribute to the growing yet still relatively limited body of literature on the benefits of working with trauma survivors, particularly through the lens of VPTG. Participants in the current study reported changes that can be conceptualized as mirroring the foundational model of PTG (Tedeschi et al., 2018). Also consistent with prior research, the current study highlights nuanced distinctions of VPTG in relation to PTG, such as the perception of personal strength being more abstract and tied to a broader sense of the human capacity to thrive. A key contribution of this study is the uncovering and suggestion of framing of VPTG as both a process and an outcome, an approach not explicitly examined in prior research. While past studies have described aspects of VPTG that suggest parallels to PTG in both its progression and its end-state, no study to date has explicitly explored both dimensions or framed findings within the overarching process and outcome framework. This study also highlights associated outcomes of VPTG, such as inspiration, compassion satisfaction, renewed hope, stronger coping skills, and better mental health, as described by participants within this specific context, underscoring its broader benefits for well-being for this specific sample group.

Our findings provide preliminary evidence that the VPTG process may parallel the PTG process in several key ways, as interpreted through participants’ accounts, characterized by cognitive and emotional struggle, deliberate meaning-making, and eventual growth and transformation. These results suggest that ITE related struggle and growth may be intertwined, and that growth and well-being may emerge through, rather than despite, distressing experiences as occurs in PTG. Likewise, the outcomes of VPTG appear to align with PTG outcomes, albeit with some nuances in the particular domains of life where growth is experienced. By examining VPTG within the occupational and relational context of peer-support, these findings expand our understanding of how personal growth may unfold in professional roles marked by ongoing exposure to trauma. These insights offer a tentative, context-bound framework for understanding how indirect trauma exposure may catalyze growth, providing direction for future research.

Beyond research implications, the findings also point to potential for practical applications for training, supervision, and organizational support systems. Programs that train and supervise helping professionals may choose to intentionally incorporate reflection-based and growth-oriented practices (e.g., structured peer dialogue, emotional regulation training, and meaning-making exercises) to support the processing of distress and promote growth. Supervisors and leaders might likewise recognize distress not only as a risk factor but also as a potential entry point for professional and personal growth when supported appropriately, especially in settings where helping professionals are trained to understand and recognize PTG and VPTG.

## Data Availability

The data presented in this study are available on request from the corresponding author due to the sensitive and potentially identifiable nature of these qualitative data.
